# The Role of Fibroblasts in Melanoma Development: From Tumor Microenvironment Remodeling to Pre-Metastatic Niche Formation

**DOI:** 10.3390/ijms26136132

**Published:** 2025-06-26

**Authors:** Immacolata Belviso, Raffaele Pastore, Aldo Mileo, Emiliano Del Genio, Stefania Boccia, Stefano Palermi, Carmine Sellitto, Maria Letizia Motti

**Affiliations:** 1 Department of Psychology and Health Sciences, Telematic University Pegaso, 80143 Naples, Italy; immacolata.belviso@unipegaso.it; 2 Department of Medicine and Health Sciences “Vincenzo Tiberio”, University of Molise, 86100 Campobasso, Italy; raffaele.pastore@unimol.it (R.P.); aldo.mileo@unimol.it (A.M.); emiliano.delgenio@unimol.it (E.D.G.); 3 Department of Mental and Physical Health and Preventive Medicine, Section of Human Anatomy, University of Campania “Luigi Vanvitelli”, 80131 Naples, Italy; stef.boccia.sb@gmail.com (S.B.); carmine.sellitto@tiscali.it (C.S.); 4 Departmental Faculty of Medicine and Surgery, UniCamillus-Saint Camillus International University of Health Sciences, 00131 Rome, Italy; stefano.palermi@unicamillus.org; 5 Department of Medical, Human Movement and Well-Being Sciences, University of Naples Parthenope, 80133 Naples, Italy

**Keywords:** fibroblast, cancer-associated fibroblasts (CAFs), pre-metastatic niche, metastasis, extra cellular matrix (ECM), melanoma, tumor microenvironment (TME)

## Abstract

Melanoma is the most aggressive form of skin cancer, and despite significant therapeutic advances over the past decade, a substantial number of patients still progress to a fatal outcome. The initiation and progression of melanoma are strongly influenced by interactions between melanoma cells and other components of the tumor microenvironment (TME). In this review, we focus on the interplay between fibroblasts resident in the tumor microenvironment and tumor cells. In particular, we examine the molecular mechanisms through which melanoma cells induce the transformation of resident fibroblasts into their active form, known as cancer-associated fibroblasts (CAFs). We also explore the role of CAFs in shaping the melanoma microenvironment (MME) and in organizing the pre-metastatic niche, a specialized microenvironment that forms in distant organs or tissues to support the survival and expansion of metastatic melanoma cells. Finally, we discuss emerging therapeutic strategies aimed at disrupting the interactions between CAFs, melanoma cells, and other components of the tumor microenvironment to improve treatment outcomes.

## 1. Introduction

It is widely recognized that melanomagenesis can result from alterations in the internal mechanisms of cancer cells, such as mutations in specific genes (*BRAF*, *CDKN2A*, *NRAS*, and *TP53*) or epigenetic alterations, including the deregulation of non-coding RNAs such as microRNAs (miRNAs) and long non-coding RNAs (lncRNAs) [1,2,3].

The development of targeted therapies for melanoma has arisen from the identification of these genetic mutations, and such therapies have shown remarkable clinical success [4,5]. In modern clinical settings, therapeutic approaches frequently involve targeting the mitogen-activated protein kinase (MAPK) signaling cascade. Drugs designed to inhibit BRAF (such as encorafenib, dabrafenib, and vemurafenib) and MEK (including trametinib, binimetinib, and cobimetinib) are widely utilized, either as monotherapy or in combination regimens [4,5].

However, many patients initially respond to these therapies, but after approximately six to eight months, develop resistance mechanisms, limiting the long-term efficacy of these treatments [6]. Over the past ten years, the treatment of melanoma has been revolutionized by the introduction of immunotherapies, particularly immune checkpoint blockade. Since their initial FDA approval in 2011, checkpoint inhibitors have become central to melanoma treatment strategies. These monoclonal antibodies inhibit key immune-regulatory molecules, including PD-1 (e.g., pembrolizumab, nivolumab), PD-L1 (e.g., atezolizumab), CTLA-4 (e.g., ipilimumab), and LAG-3 (e.g., relatlimab). Used either as monotherapy or in combination, these agents have demonstrated remarkable success in clinical trials involving advanced melanoma cases [7,8]. Although melanoma therapy has been greatly improved by the use of immune checkpoint inhibitors, not all patients respond to them, showing primary or secondary resistance [9,10].

For this reason, there is a need to identify new target molecules and molecular mechanisms that regulate tumor development, progression, and drug resistance, enabling more effective and long-lasting treatment strategies [11,12,13,14].

In this context, recent studies have highlighted complex interactions between melanoma cells and the TME, showing that they play a very important role in supporting disease progression [15]. The TME is composed of cancer cells, immune cells, stromal cells, and the extracellular matrix (ECM), all of which interact through soluble molecules released by both cancerous and non-cancerous cells [16]. The genesis of the TME is driven by stromal alterations induced by tumor development and is shaped by the dynamic balance between pro-tumorigenic and antitumor cells. These cellular populations interact to establish the specific characteristics of the TME [17,18,19].

Cells within the melanoma microenvironment (MME) produce molecules that, in a paracrine manner, affect tumor growth by degrading the extracellular matrix, enhancing melanoma angiogenesis, and increasing tumor cell invasiveness [20].

In the MME, melanoma cells lose their responsiveness to keratinocyte regulation because E-cadherin expression is replaced by N-cadherin. The extracellular domain of N-cadherin facilitates interactions with other cells that express N-cadherin, allowing melanoma cells to communicate and bind to fibroblasts [21].

Fibroblasts within the MME become activated through interactions with melanoma cells and differentiate into CAFs. These CAFs, in turn, promote tumor cell proliferation, enhance invasiveness and migratory capacity, and influence the patient’s response to therapy and prognosis [20,22,23,24,25].

In this review, we examine the role of reciprocal interactions between melanoma cells and fibroblasts in regulating tumor growth and progression. Furthermore, we discuss the potential of targeting these interactions as a promising therapeutic strategy.

## 2. Crosstalk Between Fibroblasts and Melanoma Cells in the Melanoma Microenvironment (MME)

In the MME, the reprogramming of fibroblasts to CAFs by tumor cells plays a pivotal role in tumor promotion and progression, influencing several aspects of melanoma biology. CAFs acquire a specific pro-tumorigenic phenotype, characterized by enhanced contractility, ECM production, and secretion of cytokines and chemokines [26].

**Figure 1:**
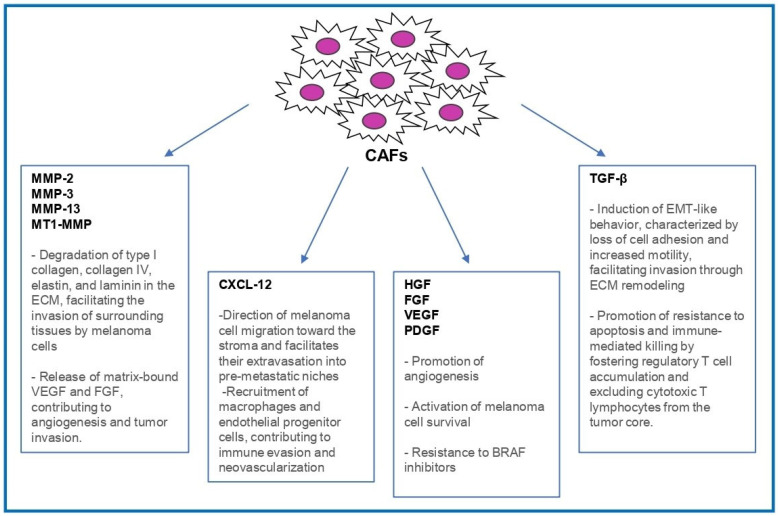
Key roles of CAFs in melanoma progression. Schematic representation of the key roles of fibroblasts activated by melanoma-derived signals in promoting tumor development and progression. Metalloproitenase (MMP), extracellular matrix (ECM), CXC motif chemokine ligand 12 (CXCL12), hepatocyte growth factor (HGF), fibroblast growth factor (FGF), vascular endothelial growth factor (VEGF), platelet-derived growth factor (PDGF), Epithelial- mesenchymal transition (EMT), transforming growth factor beta (TGF-β).

Among these, CXC motif chemokine ligand 12 (CXCL12), also known as stromal-derived factor-1) is notably enriched. CXCL12 binds to chemokine receptor type 4 (CXCR4 receptor) expressed on melanoma cells, promoting chemotaxis, survival, and homing to pre-metastatic niches such as the lungs and lymph nodes [27,28,29,30]. The functional role of the CXCL12/CXCR4 axis has been validated in preclinical models, where CXCR4 inhibition reduced both invasion and metastatic dissemination [31]. Additionally, CXCL12 mediates the recruitment of macrophages and endothelial progenitor cells, contributing to immune evasion and neovascularization [32,33,34,35] (Figure 1).

Beyond the production of chemokines, CAFs represent a crucial source of transforming growth factor beta (TGF-β), a cytokine that profoundly impacts melanoma progression. Within the primary tumor microenvironment, TGF-β acts on melanoma cells to induce changes similar to epithelial-to-mesenchymal transition (EMT), characterized by reduced cell adhesion, increased motility, and elevated expression of matrix metalloproteinases (MMPs). These alterations facilitate the remodeling of the extracellular matrix and support local tumor invasion [36].

Furthermore, TGF-β plays a significant role in modulating immune responses by promoting regulatory T cell accumulation and hindering the infiltration of cytotoxic T lymphocytes into the tumor core, thereby establishing a protective physical and immunological barrier around the tumor [37,38,39] (Figure 1).

Moreover, activated CAFs release hepatocyte growth factor (HGF), vascular endothelial growth factor (VEGF), and fibroblast growth factor 2 (FGF2), which promote angiogenesis and activate survival pathways in melanoma cells [40,41,42,43]. In *BRAF*-mutant melanomas, CAF-derived HGF has been implicated in resistance to BRAF inhibitors by reactivating the MAPK signaling pathway, thereby allowing melanoma cells to evade therapeutic pressure [24,44,45] (Figure 1).

The reciprocal communication between melanoma cells and fibroblasts is not only mediated by soluble factors but also by extracellular vesicles (EVs), particularly exosomes that deliver regulatory microRNAs (miRNAs), proteins, and metabolites [46]. These interactions contribute to the remodeling of the ECM, induce immune evasion and angiogenesis, and improve pre-metastatic niche formation with a sophisticated mechanism of stromal reprogramming [47].

Dror et al. demonstrated that miR-211 enclosed in melanosomes is transferred to dermal fibroblasts, where it downregulates the insulin-like growth factor 2 receptor (IGF2R), leading to the activation of the MAPK pathway [48]. This, in turn, promotes fibroblast proliferation, metabolic reprogramming, and the secretion of cytokines such as IL-1β, IL-6, and IL-8, thereby creating a pro-inflammatory microenvironment that precedes a visible tumor expansion [48,49].

## 3. Cancer-Associated Fibroblasts (CAFs) Activate Extracellular Matrix (ECM) Remodeling

CAFs are responsible for the extensive production and remodeling of the ECM (Figure 1). Specifically, during melanoma progression, activated fibroblasts secrete abundant structural ECM proteins, such as collagens (especially type I collagen), fibronectin, laminin, and various proteoglycans accumulating around the tumors and leading to the formation of a denser extracellular matrix, rich in collagen fibers or, in some cases, a high proteoglycan content stroma [50]. The deposition of collagen and fibronectin driven by CAFs provides a scaffold that not only enhances melanoma cell adhesion and migration but also increases tissue stiffness, which can promote the invasive behavior of tumor cells by activating a specific mechano-signaling pathway [50]. By building up a permissive fibrotic scaffold and carving out routes for dissemination, CAFs fundamentally reshape the melanoma microenvironment to support tumor invasion and metastasis. Melanoma cells are indeed highly sensitive to ECM composition, stiffness, and alignment, as they directly interact with the ECM in the tumor’s microenvironment via cell surface receptors, secreted factors, or enzymes [51,52,53]. Furthermore, PDGF released by melanoma cells has been shown to stimulate nearby fibroblasts to produce collagen, fibronectin, and laminin, illustrating how the crosstalk between tumor cells and the stromal microenvironment can prompt the remodeling of the ECM [54,55]. As a matter of fact, clinical melanoma specimens often exhibit activated fibroblasts co-expressing ECM proteins (e.g., collagen, fibronectin) and myofibroblastic markers (α-SMA), highlighting the direct role of CAFs in reshaping the tumor stromal microenvironment [56].

Additionally, to facilitate melanoma invasion, CAFs actively remodel and degrade the ECM, secreting high levels of MMPs that digest structural barriers and support tumor growth [50]. Secretion of MMPs by CAFs not only destroys physical barriers but also disengages pro-tumoral signals embedded in the ECM, amplifying melanoma progression [57]. Specific proteases like MMP-2, MMP-9, MMP-13, and MT1-MMP are often upregulated in the melanoma stromal microenvironment, where they degrade type I collagen fibers and basement membrane components like collagen IV, elastin, and laminin, operating a targeted ECM proteolysis that smooths the way for melanoma cells to invade surrounding tissue [58,59]. Moreover, MMPs activity releases bioactive molecules that are physiologically sequestered within the ECM, such as growth factors and angiogenic factors [60]. For instance, MMP-mediated collagen degradation can free VEGF and FGF bound to the matrix, thus accelerating new blood vessel formation, and, consequently, tumor invasion [61]. High MMP expression in melanoma lesions correlates with advanced disease and poorer patient survival [62], highlighting the pathological importance of ECM remodeling driven by CAFs.

## 4. Fibroblasts in the Pre-Metastatic Niche

The early stages of the metastatic process are characterized by a hostile microenvironment for the engraftment of tumor cells in distant organs. Fibroblasts in distant organs (e.g., in the lungs, liver, or bone) can be reprogrammed by circulating melanoma cells to create a favorable environment for metastatic growth. Indeed, the characterization of melanoma-derived exosomes has demonstrated their function in promoting metastatic progression [63] by reprogramming stromal cells and promoting the formation of an inflammatory metastatic niche that promotes tumor development and suppresses adaptive immunity [64].

Distinctly, at distant metastatic sites, melanoma-derived TGF-β stimulates resident fibroblasts to produce extracellular matrix components such as collagen and tenascin-C, which contribute to the formation of a pre-metastatic niche that supports subsequent tumor colonization [50].

In turn, the reprogramming of stromal cells induces a modification of the microenvironment that begins to favor the colonization of the organ and metastatic growth [65,66]. The fibroblasts present in secondary sites remodel the extracellular matrix in the pre-metastatic niche, acting as a true guide for the tumor cells; they create paths through the action of forces and proteases [67]. Force-induced matrix remodeling is activated by α3 and α5 integrins and by activation of the Rho pathway in fibroblasts [67]. Furthermore, infiltrating tumor cells, in turn, stimulate fibroblasts of secondary target organs to express periostin, a component of the stromal extracellular matrix that promotes colonization by tumor cells [68]. Moreover, fibroblasts contribute to the formation of new blood vessels, a crucial process for metastatic growth. The VEGF and FGF secreted by fibroblasts can induce vascularization at the metastatic site, providing nutrients and oxygen to the growing melanoma metastases [69,70,71]. EVs secreted by melanoma cells contain several miRNAs, including miR-155. By regulating the suppressor of cytokine signaling type 1 (SOCS1), miR-155 activates the Janus kinase 2/STAT3 signaling pathway, promoting the expression of pro-angiogenic factors (VEGFα, FGF2, and MMPs) in CAFs [72].

Similar to what occurs in the primary tumor microenvironment, fibroblasts located at metastatic sites also contribute to shaping an immunosuppressive milieu that facilitates melanoma’s immune evasion. In these pre-metastatic niches, fibroblasts may secrete TGF-β, which suppresses the activation of immune effector cells such as T lymphocytes and natural killer (NK) cells, thereby enabling melanoma cells to evade immune-mediated destruction [73].

## 5. Therapeutic Implications

Given the significant role of fibroblasts in the development of the primary tumor and the metastatic niche in melanoma progression, targeting CAFs or ECM components and secreted products may represent a promising strategy to disrupt the tumor microenvironment and enhance therapy [72]. Table 1 summarizes the potential therapeutic targets and the studies conducted to investigate them.

### 5.1. Inhibition of CXCR4

Further, in melanoma cells, CXCR4 is expressed on fibroblasts and CD133^+^ melanoma subpopulations. This signaling axis regulates tumor angiogenesis and immune cell trafficking within the TME [74,75,76], with evidence showing CXCL12’s chemotactic action on CD133^+^ cells [77]. The inhibition of CXCR4 has also been linked to suppressed melanoma growth and enhanced CD8^+^ T cell infiltration in in vivo models [27,78,79]. In a phase Ib clinical trial involving patients with advanced metastatic melanoma, Andtbacka et al. reported that treatment with the CXCR4 inhibitor mavorixafor for three weeks enhanced the recruitment of CD8^+^ T cells into melanoma lesions. Immunohistochemical analysis of patient biopsy samples revealed a marked increase in CD8^+^ T cells at the interface between tumor and adjacent normal tissue, compared to controls [80]. Collectively, these findings indicate that mavorixafor facilitates immune cell infiltration into melanoma metastatic sites. The enhanced infiltration of CD8^+^ T cells into the TME has been associated with reduced metastatic potential and improved therapeutic outcomes in melanoma [81,82,83,84].

### 5.2. Inhibition of Fibroblast Activation Protein (FAP)

Fibroblast activation protein (FAP) is a transmembrane serine protease classified within the dipeptidyl peptidase (DPP) family. It is predominantly expressed by CAFs and is detected on stromal fibroblasts of all melanocytic tumors, including benign, premalignant, and malignant lesions [16,85]. The marked upregulation of FAP is widely regarded as a defining marker of CAFs and has been associated with poor prognosis across multiple cancer types [86,87,88]. Capaccione et al. evaluated a novel therapeutic approach for melanoma, based on the targeting of fibro-blast activation protein (FAP) with the radionuclide ^177^Lu-FAPI-04. To this end, B16F10 murine models of melanoma were treated with ^177^Lu-FAPI-04 alone or in combination with checkpoint inhibitor immunotherapy (anti-murine CTLA-4 and anti-murine PD-1). Notably, both monotherapy with ^177^Lu-FAPI-04 and combined radioligand-immunotherapy induced tumor regression, with the combined approach showing increased tumor cell apoptosis and reduced proliferative indices. Flow cytometric analysis revealed distinct, tumor-specific changes in tumor-associated macrophages in the combination therapy group, differing from the effects seen with either treatment alone. These findings highlight the potential of ^177^Lu-FAPI-04 as an effective therapeutic strategy for melanoma, working at least in part through the induction of apoptosis and modulation of the tumor immune microenvironment. Further translational studies are warranted to evaluate the clinical potential of this combination regimen. Collectively, these findings support the therapeutic utility of ^177^Lu-FAPI-04 as a monotherapy and its potential to enhance the efficacy of immunotherapy through complementary mechanisms involving direct cytotoxicity and remodeling of the tumor immune microenvironment [89]. In a phase Ib clinical study, Munoz-Cosuelo et al. evaluated the combination of fibroblast activation protein interleukin-2 variant (FAP-IL2v), a novel immunocytokine engineered to address the limitations of wild-type IL-2, with the checkpoint inhibitor pembrolizumab in patients with advanced or metastatic melanoma, including both checkpoint inhibitor (CPI)-naïve and CPI-experienced individuals. The combination therapy demonstrated limited antitumor efficacy in patients who had previously progressed on CPI treatment, indicating that FAP-IL2v alone is insufficient to overcome resistance or lack of responsiveness to checkpoint inhibition [90]. Talabostat is an orally bioavailable inhibitor of dipeptidyl peptidases with immunostimulatory properties that demonstrated antitumor activity in a mouse model of melanoma [91]. Nevertheless, results from the Eager et al. phase II clinical trial showed that the combination of talabostat and cisplatin in patients with metastatic melanoma did not improve anticancer activity in comparison to standard of care therapies [92].

### 5.3. Inhibition of TGF-β

Beyond its well-known immunosuppressive and pro-invasive functions, TGF-β exhibits additional context-dependent roles during melanoma progression. While initially acting as a growth inhibitor in normal melanocytes and early-stage lesions, its signaling undergoes a functional switch in malignancy, becoming a critical driver of tumor progression [54]. In this pathological setting, melanoma cells exploit TGF-β signaling to suppress the production of key immunomodulatory cytokines, such as TNF-α, VEPH1, and INF-γ, and to alter regulatory pathways including Notch1, IL-6, and ERK/MAPK, ultimately promoting an immunologically inert microenvironment [93,94,95].

Moreover, excessive TGF-β expression hampers interleukin-12-mediated stimulation of CD4^+^, CD8^+^ T cells, and NK cells, impairing both lymphocyte proliferation and their cytotoxic activity, thereby contributing to immune escape [96,97].

Due to this dual role in promoting tumor progression and subverting immune responses, therapeutic strategies aimed at neutralizing TGF-β signaling have gained significant attention. Preclinical studies in melanoma models, such as those employing the TGF-β receptor inhibitor SB-505124 in combination with IL-12-based immunotherapy, have shown promising results in enhancing antitumor efficacy and prolonging survival [96,98]. Among the emerging agents, Fresolimumab (GC1008), a monoclonal antibody targeting all isoforms of TGF-β, represents a potential candidate for clinical application. In a phase I clinical trial, Morris et al. assessed the safety and antitumor activity of GC1008 in patients with advanced malignant melanoma. The study found that repeated dosing was generally well tolerated and showed preliminary signs of antitumor efficacy. These findings support continued investigation of GC1008 as both a monotherapy and in combination with other anticancer agents [99].

**Table 1:** Potential therapeutic targets in melanoma microenvironment-directed therapy.

Targets	Inhibitory Molecules	Drug Class	Phase of Clinical Studies	Clinical Trial	Current Status of the Clinical Trial	Refs.
CXCR4	CXCR4 antagonist X4-136	Small molecules	Preclinical			[73]
	CXCR4 antagonist Mavorixafor		Clinical phase I	NCT02823405	Not yet completed, completion in 2029	[80]
FAP	^177^Lu-FAPI-04 (radionuclide)	Fibroblast Activation Protein (FAP) inhibitors	Preclinical			[85]
	Simlukafusp alfa, RO6874281 (FAP-IL2v)	Engineered immunocytokine	Clinical phase I	NCT02627274	Completed. Tolerability acceptable; common adverse events included fatigue, asthenia, and drug-induced liver injury	[90]
	Talabostat	Small molecules	Clinical phase II	NCT00083239	Completed, limited efficacy as monotherapy, highlighting the need for further studies, potentially in combination with other agents	[92]
TGF-β	SB-505124 + IL-12	Small molecule+ encoding adenoviral vector	Preclinical			[96,98]
	Fresolimumab	Human monoclonal antibodies	Clinical phase I	NCT00356460	Completed. Well-tolerated with manageable adverse events; limited efficacy data but supports further TGF-β research	[99]
MMPs	Batimastat	Angiogenesis inhibitors	Preclinical			[100,101]
	Marimastat	Small molecules	Clinical phase II	NCT00004248	Completed, limited clinical benefit and significant side effects, leading to discontinuation of the trial for melanoma	[102]

### 5.4. Inhibition of MMPs

Matrix metalloproteinases (MMPs) have been extensively studied for their role in melanoma progression, particularly in facilitating extracellular matrix degradation, tumor invasion, and metastasis. This has led to the development of MMP inhibitors (MMPIs) such as Batimastat and Marimastat. Batimastat, a broad-spectrum MMPI, demonstrated efficacy in preclinical models by inhibiting tumor growth and angiogenesis. However, its clinical application was limited due to poor solubility and low oral bioavailability, necessitating intraperitoneal administration, which posed practical challenges and led to adverse effects like peritonitis and liver dysfunction. [100,101].

Marimastat, developed as an orally bioavailable analog, showed promise in early-phase clinical trials. Nevertheless, its non-selective inhibition of MMPs and related enzymes such as ADAMs resulted in significant side effects, including musculoskeletal pain, inflammation, and fibrosis. These adverse events, coupled with a lack of substantial survival benefits, led to the discontinuation of Marimastat’s development. However, in a phase II trial involving patients with metastatic melanoma, Marimastat demonstrated only modest therapeutic efficacy [100,101,102].

The challenges faced by MMPIs underscore the complexity of targeting proteolytic enzymes in cancer therapy. MMPs not only facilitate tumor progression but also play roles in normal physiological processes, such as tissue remodeling and the release of anti-angiogenic factors. Inhibiting these enzymes indiscriminately can disrupt these processes, leading to unintended consequences [101].

Beyond MMPs, other proteolytic enzymes like cathepsins and urokinase-type plasminogen activator (uPA) have been implicated in melanoma progression. Cathepsins B, K, and L, as well as uPA and its receptor uPAR, are upregulated in melanoma and stromal cells, contributing to tumor invasiveness. While these enzymes present potential therapeutic targets, none have yet advanced to clinical trials specifically for melanoma [103,104,105].

In conclusion, while proteolytic enzymes remain attractive targets in melanoma therapy, the development of effective and safe inhibitors requires a nuanced understanding of their diverse roles in both tumor biology and normal physiology.

### 5.5. Additional TME-Related Targets in Melanoma Treatment

In addition to CAFs, ECM components, and previously discussed targets such as CXCR4, FAP, TGF-β, and MMPs, several other TME-associated molecules and pathways have emerged as relevant to melanoma progression and therapy resistance. These include the vitamin D receptor (VDR), platelet-derived growth factor receptor (PDGFR), fibroblast growth factor receptor (FGFR), angiotensin receptor signaling, JAK-STAT signaling, the Hedgehog (Hh) pathway, and hyaluronic acid.

The VDR, expressed in both melanoma cells and stromal fibroblasts, has been implicated in regulating cell proliferation, differentiation, and immune modulation. Its activation may suppress melanoma progression and enhance immune surveillance [106,107].

Angiotensin receptor signaling, particularly via the angiotensin II type 1 receptor (AT1R), has been linked to immunosuppression and fibrosis within the TME. Targeting this axis may improve vascular normalization and drug delivery [108,109,110,111]. Similarly, the JAK-STAT pathway plays a role in stromal inflammation and immune evasion, and its pharmacological blockade may restore antitumor immunity [112].

The Hedgehog pathway, involved in tissue patterning and fibroblast behavior, is aberrantly activated in various tumors and supports CAF expansion and ECM production [113]. Finally, hyaluronic acid, a major ECM component, influences melanoma cell motility and immune exclusion. Strategies to degrade or block its accumulation are under investigation to improve TME accessibility and therapy penetration [114,115].

Together, these stromal-related targets represent promising avenues for combinatorial strategies aimed at disrupting TME-mediated resistance mechanisms in melanoma.

## 6. Conclusions and Discussion

This review explores the role of interactions between fibroblasts and melanoma cells within the tumor microenvironment (TME) in driving tumor initiation, progression, and the formation of the pre-metastatic niche. Previous studies have demonstrated that genetic and epigenetic alterations in melanoma cells are key oncogenic drivers, enabling the development of targeted therapies that are still used in the treatment of melanoma [4,5]. However, responses to these therapies are often not durable, highlighting the need for continued research to achieve long-lasting treatment outcomes.

In recent years, growing evidence has indicated that the interaction between tumor cells, the stroma, and the extracellular matrix is crucial for the genesis and progression of melanoma. A dynamic and not yet fully understood balance exists within the TME, where stromal cells, tumor cells, and the extracellular matrix influence one another through complex feedback loops [14,15,16,17].

Within this environment, fibroblasts can transition from an inactive to an active state, becoming CAFs. These CAFs, in turn, stimulate tumor cells and contribute to melanoma progression [20,22,23,24,25]. Biomedical research is increasingly focusing on the TME to identify potential therapies that target the multiple interactions occurring within it.

The ultimate goal of this review is to highlight how the complex interactions between fibroblasts and tumor cells can play a crucial role in clinical outcomes. A deeper understanding of the alterations in tumor cells and their crosstalk with other components of the TME, particularly fibroblasts, may offer new targets for therapeutic intervention. CAFs have emerged as promising targets for anticancer therapies; however, their clinical application is challenged by several intrinsic complexities. A primary difficulty arises from their heterogeneous cellular origins, which vary depending on the tissue and tumor microenvironment. CAFs exhibit substantial phenotypic and functional heterogeneity within the TME, where they originate from various sources, including tissue-resident fibroblasts, mesenchymal stem cells, endothelial cells, and pericytes. Advances in single-cell RNA sequencing and proteomic profiling have refined the classification of CAF subsets, revealing transcriptionally distinct populations that vary according to tumor context and evolve dynamically during disease progression [116].

Among the primary CAF subtypes are myofibroblastic CAFs (myCAFs), which are implicated in extracellular matrix (ECM) remodeling and collagen deposition, processes that contribute to tissue stiffness, tumor growth, and metastatic dissemination; inflammatory or immune-modulatory CAFs (iCAFs), which influence immune cell recruitment, suppress antitumor immunity, and support tumor cell survival and proliferation; and antigen-presenting CAFs (apCAFs), a less-characterized group thought to engage with T lymphocytes, potentially shaping local immune responses [116] (Table 2).

These diverse subsets underscore the dualistic and sometimes opposing roles of CAFs in modulating tumor architecture and immune dynamics. Their complexity necessitates a nuanced understanding when designing targeted therapies, as different CAF populations may either support or hinder therapeutic efficacy [116].

Despite advances in identifying CAF-associated markers, no single biomarker demonstrates absolute specificity. Currently, CAFs are defined by the lack of epithelial, endothelial, and hematopoietic markers, while expressing mesenchymal markers such as vimentin, alpha-smooth muscle actin (α-SMA), fibroblast activation protein (FAP), and platelet-derived growth factor receptor alpha (PDGFRα), typically without oncogenic mutations [117,118]. Due to this complexity, accurate identification of CAFs necessitates the combined use of multiple markers adapted to the specific tumor context.

However, molecular data alone are not sufficient; interpreting the functional roles of each CAF subset in relation to tumor cells, immune infiltrates, and other stromal components is equally important. The classification of CAF subtypes emphasizes not only their phenotypic variability but also their ability to adapt functionally to changes within the tumor microenvironment. In particular, the subset known as inflammatory CAFs (iCAFs) has been shown to foster an immunosuppressive environment through the expression of immune checkpoint ligands, including PD-L1 and PD-L2, which inhibit T cell activity without requiring direct cell contact. Additionally, factors secreted by CAFs, such as CXCL2, CXCL5, or miRNAs delivered via extracellular vesicles, can stimulate PD-L1 expression in tumor cells, further limiting the effectiveness of immune responses. Notably, certain CAF populations have also been reported to display antigen-presenting features and to trigger antigen-specific cytotoxic T cell death, highlighting the extent to which CAF heterogeneity impacts immune regulation and resistance to immune checkpoint therapies [119].

Clarifying the relationships among CAF subsets, whether they function as independent lineages, occupy overlapping states, or follow a hierarchical structure, is crucial. Mapping these interactions and spatial arrangements within the tumor microenvironment could enable the development of more precise therapeutic strategies aimed at targeting only the CAF populations that actively contribute to tumor progression, while preserving or even enhancing those that may exert regulatory or antitumoral effects.

## Figures and Tables

**Table 2:** CAFs comprise heterogeneous subtypes with distinct origins and functions within the TME. Among the main categories are myofibroblastic CAFs (myCAFs), inflammatory CAFs (iCAFs), and antigen-presenting CAFs (apCAFs).

CAFs Subtypes	Functions in the TME
Myofibroblastic CAFs (myCAFs)	involved in extracellular matrix remodeling and collagen deposition, contributing to increased tissue stiffness, tumor progression, and metastasis
Inflammatory CAFs (iCAFs)	secrete cytokines and chemokines that modulate the immune response, promoting immune evasion, cancer cell survival, and proliferation
Antigen-presenting CAFs (apCAFs)	characterized by the expression of MHC class II molecules, they may interact with T cells, although their immunological role is still being clarified

## Data Availability

No new data were created or analyzed in this study.

## Abbreviations

CAF	cancer-associated fibroblast
TME	tumor microenvironment
MME	melanoma microenvironment
CXCR4	chemokine receptor type 4
CXCL12	CXC motif chemokine ligand 12
SDF1	stromal-derived factor-1
TGF-β	transforming growth factor beta
EMT	epithelial-mesenchymal transition
HGF	hepatocyte growth factor
VEGF	vascular endothelial growth factor
FGF2	fibroblast growth factor 2
IGF2R	insulin-like growth factor 2 receptor
MAPK	mitogen-activated protein kinase
EV	extracellular vesicle
miRNA	Micro-RNA
IL	interleukin
FAP	Fibroblast activation protein
DPP	dipeptidyl peptidase
IFN-γ	interferon-gamma
TNF-α	tumor necrosis factor-alpha
VEPH1	Ventricular Zone Expressed PH Domain Containing 1
SMAD4	Mothers against decapentaplegic homolog 4
SKI	subtilisin/kexin isozyme
myCAFs	Myofibroblastic CAFs
iCAFs	inflammatory CAFs
apCAFs	Antigen-presenting CAFs
VDR	vitamin D receptor
PDGFR	platelet-derived growth factor receptor
FGFR	fibroblast growth factor receptor
Hh	Hedgehog
AT1R	angiotensin II type 1 receptor
